# Gene Set Signature of Reversal Reaction Type I in Leprosy Patients

**DOI:** 10.1371/journal.pgen.1003624

**Published:** 2013-07-11

**Authors:** Marianna Orlova, Aurélie Cobat, Nguyen Thu Huong, Nguyen Ngoc Ba, Nguyen Van Thuc, John Spencer, Yohann Nédélec, Luis Barreiro, Vu Hong Thai, Laurent Abel, Alexandre Alcaïs, Erwin Schurr

**Affiliations:** 1 McGill International TB Centre, The Research Institute of the McGill University Health Centre, Montreal, Quebec, Canada; 2 Departments of Human Genetics and Medicine, McGill University, Montreal, Quebec, Canada; 3 Hospital for Dermato-Venereology, Ho Chi Minh City, Vietnam; 4 Department of Microbiology, Immunology and Pathology, College of Veterinary Medicine & Biomedical Sciences, Colorado State University, Fort Collins, Colorado, United States of America; 5 Department of Pediatrics, Sainte-Justine Hospital Research Centre, University of Montreal, Montreal, Quebec, Canada; 6 Laboratory of Human Genetics of Infectious Diseases, Necker Branch, Institut National de la Santé et de la Recherche Médicale, Paris, France; 7 University Paris Descartes, Imagine Institute, Paris, France; 8 St. Giles Laboratory of Human Genetics of Infectious Diseases, Rockefeller Branch, The Rockefeller University, New York, New York, United States of America; 9 URC-CIC, Hopital Tarnier, Paris, France; Ospedale San Pietro FBF, Italy

## Abstract

Leprosy reversal reactions type 1 (T1R) are acute immune episodes that affect a subset of leprosy patients and remain a major cause of nerve damage. Little is known about the relative importance of innate versus environmental factors in the pathogenesis of T1R. In a retrospective design, we evaluated innate differences in response to *Mycobacterium leprae* between healthy individuals and former leprosy patients affected or free of T1R by analyzing the transcriptome response of whole blood to *M. leprae* sonicate. Validation of results was conducted in a subsequent prospective study. We observed the differential expression of 581 genes upon exposure of whole blood to *M. leprae* sonicate in the retrospective study. We defined a 44 T1R gene set signature of differentially regulated genes. The majority of the T1R set genes were represented by three functional groups: i) pro-inflammatory regulators; ii) arachidonic acid metabolism mediators; and iii) regulators of anti-inflammation. The validity of the T1R gene set signature was replicated in the prospective arm of the study. The T1R genetic signature encompasses genes encoding pro- and anti-inflammatory mediators of innate immunity. This suggests an innate defect in the regulation of the inflammatory response to *M. leprae* antigens. The identified T1R gene set represents a critical first step towards a genetic profile of leprosy patients who are at increased risk of T1R and concomitant nerve damage.

## Author Summary

Leprosy type 1 reversal reactions (T1R) are an important cause of nerve damage in leprosy patients and accurate prediction of patients at increased risk of T1R is a major challenge of current leprosy control. The incidence of T1R differs widely from 6% to 67% of leprosy patients in different leprosy endemic settings. Whether or not this reflects the impact of unknown environmental triggers or differences in the genetic background across ethnicities is not known. We performed a comparative transcriptome analysis between leprosy patients affected and free of T1R in response to *M. leprae* antigens. As the discovery sample we enrolled cured leprosy patients who had been diagnosed with T1R at the time of leprosy diagnosis and leprosy patients who had never undergone T1R (retrospective arm). Whole genome transcriptome analysis after stimulation of blood with *M. leprae* antigen resulted in the definition of a T1R signature gene set. We validated the T1R gene set in RNA samples obtained from T1R-free patients at the time of leprosy diagnosis and followed for 3 years for development of T1R (prospective arm). These results confirm the role of innate factors in T1R and are a first step towards a predictive genetic T1R signature.

## Introduction

Leprosy is a chronic human infectious disease caused by *Mycobacterium leprae*. If left untreated the disease results in pronounced skin deformities and nerve disabilities due to preferential invasion of macrophages and Schwann cells by *M. leprae*. Efforts by the World Health Organisation (WHO) to eliminate leprosy resulted in a substantial reduction of global disease prevalence from 5.35 million in 1985 to 211,903 by 2010. The number of newly registered cases, however, remained at high rates (244,796 in 2009) [1]. Leprosy displays a wide spectrum of clinical manifestations. Tuberculoid (TT) and lepromatous leprosy (LL), characterized by the presence and absence of specific cellular immune responses, respectively, represent the opposite ends of the clinical spectrum [2], [3]. Based on histopathological, immunological, bacteriological, and clinical criteria, Ridley and Jopling classified three additional intermediate, or “borderline,” types as borderline tuberculoid (BT), mid borderline (BB), and borderline lepromatous (BL) leprosy [4].

Leprosy reactions, acute episodes of dysregulated inflammation, are a major cause of nerve damage in leprosy patients and present as two types [5], [6]. Type-2 reactions remain rather infrequent (<5% of leprosy patients) and occur nearly exclusively in BL and LL patients [7]. In contrast, reversal reactions type-1 (T1R) can occur in any leprosy subtype, although they are most prevalent in the borderline forms (BT-BB-BL) [8]. T1R are characterized by sudden episodes of exacerbated local delayed-type hypersensitivity to *M. leprae* in skin and/or nerves. Histological assessment of T1R lesions demonstrated an influx of mononuclear cells that lead to skin swelling and neural compression [9], [10]. Immunological analysis of the skin lesions and peripheral blood samples of patients with T1R showed the predominance of CD4^+^ T cells and Th1-associated cytokines, especially IFN-γ, IL-2, IL-12, and TNF-α [11]–[15]. The clinical care and management of T1R patients is a major challenge of current leprosy control efforts.

In Vietnam, prevalence of T1R is 29% among leprosy patients and approximately one third of T1R cases are detected at the time of leprosy diagnosis [8]. However, the prevalence of T1R differs widely among different geographical and epidemiological settings and ranges from 6% to 67% of all patients with leprosy [7], [16]–[19]. It is not known if this large spread in the occurrence of T1R reflects the variable impact of environmental triggers or an innate predisposition of certain leprosy patients towards T1R. In the absence of acute T1R, we compared the transcriptome response to *M. leprae* sonicate of leprosy patients that developed T1R with those that did not. In the discovery set we employed a retrospective design (patients who present at leprosy diagnosis with T1R) and we validated the results obtained in a prospective study (patients who present with T1R after diagnosis of leprosy). This design allowed us to identify a T1R gene set signature that captures differences in gene expression of whole blood following exposure to *M. leprae* antigens that are characteristic for persons with an innate predisposition to undergo T1R.

## Results

### Study overview

**Figure 1:**
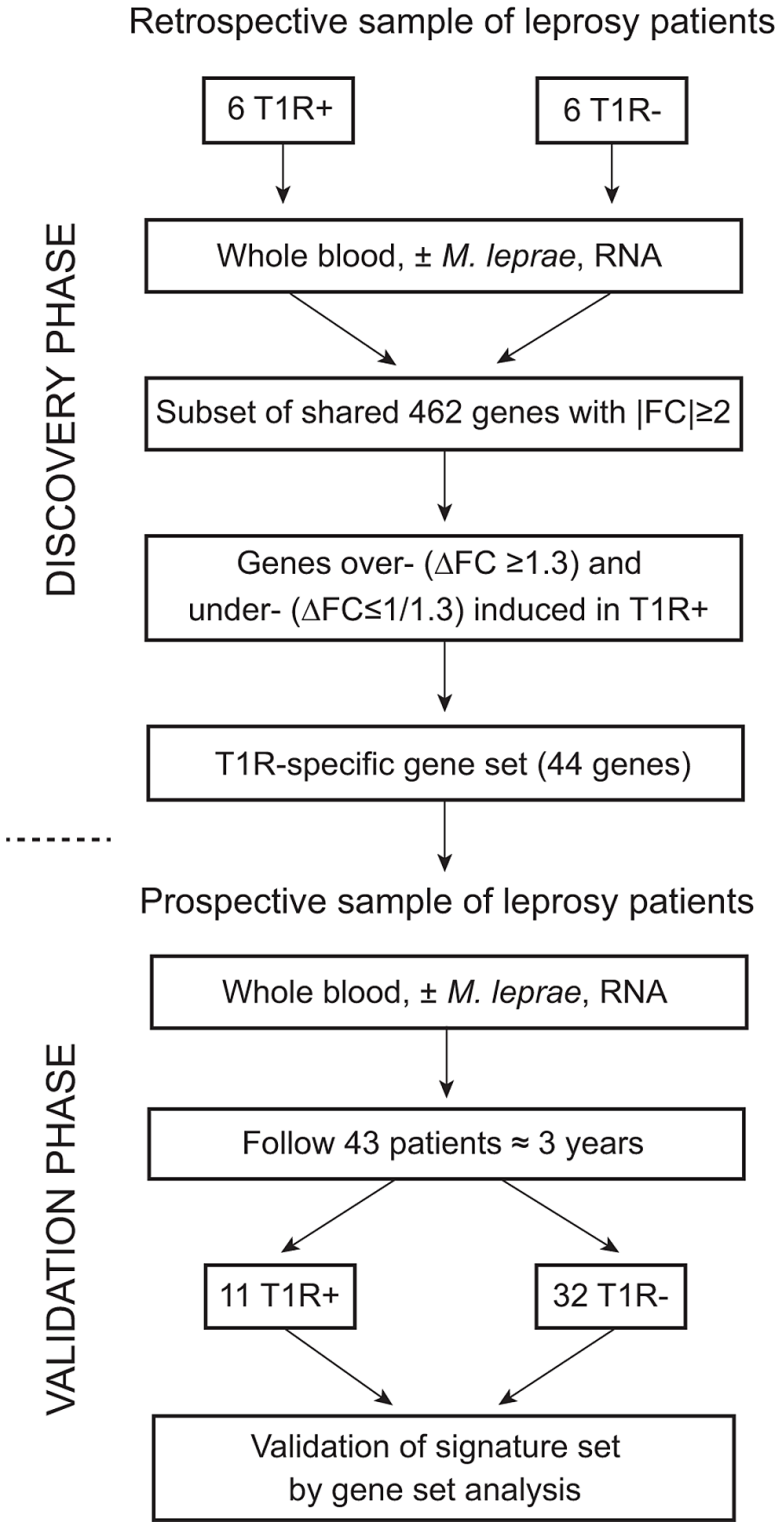
Work flow of the study. The study was subdivided into a discovery and a validation phase. In the discovery phase, we enrolled 12 leprosy patients of which six had developed T1R. All subjects had been cured of disease at least five years prior to being enrolled in the study. Whole blood of all subjects was subjected to *M. leprae* sonicate stimulation and 462 genes with an absolute fold change (|FC|)≥2 of gene expression were identified. From this set of genes we derived a subset of genes that were over- (ΔFC≥1.3) or under-stimulated (ΔFC≤1/1.3) in T1R patients. This subset of genes was termed the T1R-specific gene set. In the validation phase, we enrolled 43 leprosy patients and obtained RNA from whole blood assays at the time of enrolment. We followed these patients for at least 3 years and recorded 11 instances of T1R. We conducted a gene set analysis and validated the 44 gene T1R set in this group of T1R patients.

Our study followed a two-step design (Figure 1). First, we enrolled a retrospective sample of 12 former leprosy patients of which half had remained T1R-free while the other six had been diagnosed with T1R at the time of leprosy diagnosis. For these patients, irrespective of T1R, the time from clinical cure to participation in the present study was on average nine years [range 5–13 years]. The patients with simultaneous diagnosis of T1R and leprosy can be considered as early-onset T1R and we hypothesized that genetic effects should be most pronounced in such patients. By comparing *M. leprae* sonicate triggered gene expression in whole blood between the two groups (i.e. T1R-positive and T1R-free), we derived a gene set that was either over- or under-stimulated among the T1R group (Figure 1). Next, we employed the genes that were differentially induced (the so-called T1R-specific gene set) in a prospective design. We enrolled 43 leprosy patients who were T1R-free at the time of leprosy diagnosis and obtained RNA from whole blood assays stimulated with *M. leprae* sonicate. We then followed these patients for 3 years and recorded episodes of T1R among 11 patients. At that point, we conducted an analysis that validated the T1R gene set in the prospective arm (Figure 1). Since none of the subjects in the prospective phase had developed clinical signs of T1R at the time of the experiment, this validation showed that the T1R-specific set captured an innate characteristic of T1R susceptibility.

### Strong regulation of innate immunity genes by *M. leprae* antigens

In the retrospective samples, we conducted a careful transcriptional response profile of whole blood to *M. leprae* sonicate. Independent of the clinical phenotype, a strong transcriptional response to *M. leprae* antigens was observed in subjects of both groups. Antigen stimulation altered the expression of 581 genes by at least two-fold in at least one group. Of these, 462 genes showed the same direction and strength of regulation (absolute fold-change equal or above 2) in both groups. Even though not all of the remaining 134 genes reached the two-fold regulation cut-off in each patient group, these genes showed identical direction of *M. leprae*-triggered expression changes in both groups. The comparison of the baseline (unstimulated) and antigen-stimulated expression levels between T1R-free andT1R-affected patients did not detect significant differences in gene induction or suppression (data not shown).

We utilized the DAVID analysis tool to assess the enrichment of particular functional clusters among the 581 regulated genes. Out of 581 genes, 572 were annotated by DAVID for further analysis. The DAVID Functional Annotation Clustering tool assigns each gene to a set of functional groups according to its role in cellular processes [20]–[22]. To avoid listing multiple sources for clustered functional entities we focused on GO terms and KEGG/PANTHER pathways. As significance thresholds we used p-values adjusted by the Benjamini-Hochberg procedure (*P*_BH_). For GO terms we selected *P*_BH_<10^−5^ and for pathways *P*_BH_<0.05 with the false-discovery rate fixed at 0.05 in each instance. The highest levels of significance were observed for GO terms “Defense response” (*P*_BH_ = 6.2×10^−24^), “Inflammatory Response” (*P*_BH_ = 1.6×10^−21^) and “Response to wounding” (*P*_BH_ = 1.44×10^−20^; Table 1). More specific and still highly significant terms highlighted the overabundance of genes involved in innate immunity (e.g. “Vacuole”, “Regulation of immune system process”, “Response to bacterium”, “Cytokine activity”, “Chemotaxis”, “Regulation of cell death”, “Regulation of cell proliferation”; Table 1). Consistent with the observed GO terms, we also detected significant enrichment of three signalling pathways implicated in lysosomal function, cytokine and chemokine signalling cascades (Table 2). Taken together, these data showed a strong innate immune response to *M. lepra*e antigens irrespective of a history of T1R.

**Table 1:** Overrepresented Gene Ontology terms amongst the 572 genes regulated by *M. leprae* sonicate in the samples from former leprosy patients with and without T1R.

GO terms	Number of genes^a^	% input^b^	*P*-value^c^	*P*_BH_-value^d^
Defense response	81	14.2	8.28×10^−27^	6.20×10^−24^
Inflammatory response	57	9.9	1.15×10^−24^	1.60×10^−21^
Response to wounding	70	12.2	3.84×10^−23^	1.44×10^−20^
Response to other organism	46	8.0	2.66×10^−18^	6.64×10^−16^
Vacuole	41	7.1	8.90×10^−18^	2.40×10^−15^
Regulation of immune system process	50	8.7	4.22×10^−16^	8.30×10^−14^
Response to bacterium	33	5.7	4.25×10^−14^	5.29×10^−12^
Regulation of response to stimulus	50	8.7	6.44×10^−13^	6.02×10^−11^
Cytokine activity	31	5.4	2.75×10^−12^	5.53×10^−10^
Response to lipopolysaccharide	20	3.5	5.82×10^−12^	1.62×10^−9^
Regulation of cell death	66	11.5	3.32×10^−11^	2.46×10^−9^
Chemotaxis	26	4.5	1.04×10^−10^	7.05×10^−9^
Regulation of cell proliferation	62	10.8	4.56×10^−10^	2.44×10^−8^
Regulation of immune response	30	5.2	4.29×10^−10^	2.47×10^−8^
Response to virus	21	3.7	4.24×10^−10^	2.65×10^−8^
Regulation of cytokine production	26	4.5	1.51×10^−9^	7.04×10^−8^
Regulation of response to stress	32	5.6	2.46×10^−9^	1.08×10^−7^
Extracellular space	55	9.6	1.22×10^−9^	1.97×10^−7^
Regulation of cell activation	24	4.2	1.80×10^−8^	6.13×10^−7^

a Number of genes assigned to one GO term or pathway.

b Percentage of genes assigned to one GO term or pathway from the total number of processed genes.

c *P*-value derived from a modified Fisher's exact test.

d *P*-value corrected for multiple testing by Benjamini-Hochberg correction.

**Table 2:** Molecular pathways overrepresented amongst the 572 genes regulated by *M. leprae* sonicate in the samples from former leprosy patients with and without T1R.

Pathway source: name	Number of genes^a^	% input^b^	*P*-value^c^	*P*_BH_-value^d^
KEGG: Lysosome	22	3.8	1.64×10^−7^	1.22×10^−5^
KEGG: Cytokine-cytokine receptor interaction	32	5.6	3.28×10^−6^	1.63×10^−4^
KEGG: Chemokine signaling pathway	25	4.3	1.15×10^−5^	3.44×10^−4^

a Number of genes assigned to one pathway.

b Percentage of genes assigned to one GO term or pathway from the total number of processed genes.

c *P*-value derived from a modified Fisher's exact test.

d *P*-value corrected for multiple testing by Benjamini-Hochberg correction

### T1R gene-set signature: differential intensity of gene expression changes between T1R-affected and T1R-free leprosy patients

Although there were no significant T1R-specific differences in induction or suppression of individual transcripts, we were still interested to evaluate the T1R-specific differences in the intensity of response to *M. leprae* antigen. To quantitate differences in the intensity of the transcriptional response, we defined the delta fold change (ΔFC) value. This value captured differences in the *M. leprae* antigen triggered FC of gene expression between the groups of T1R-affected and T1R-free patients.

**Figure 2:**
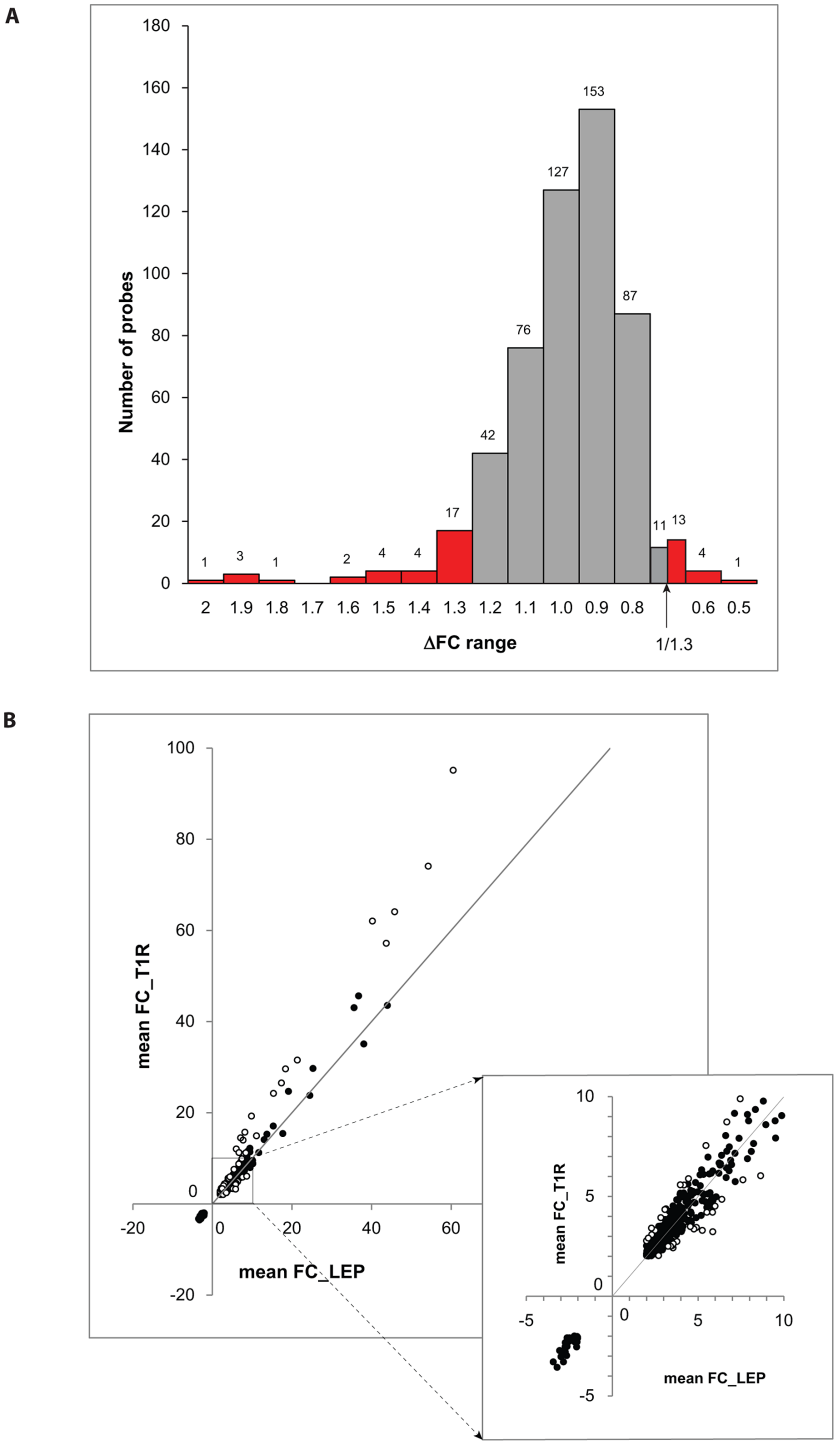
Differentially triggered genes by *M. leprae* sonicate in whole blood of T1R-affected and T1R-free leprosy patients. **A**: The distribution of ΔFC values of 532 gene probes (corresponding to 462 genes) that were induced by *M. leprae* antigen with |FC|≥2. Probes outside the cut-off values of ΔFC≥1.3 and ΔFC≤1/1.3 are indicated in red. These 50 probes which represent the extreme decile (9.4%) of the ΔFC values tagged 44 genes. These 44 genes are the T1R-specific gene set. **B**: Mean fold changes of the 532 |FC|≥2 gene probes for T1R-free leprosy patients (mean FC_LEP) plotted against the mean fold changes of the same probes for T1R-affected leprosy patients (mean FC_T1R). Each dot represents a single gene. Open circles represent the T1R gene set. The insert displays genes with mean FC of −5 to +10.

We considered a gene to be differentially regulated if its ΔFC was greater than 1.3 or smaller than 1/1.3. The justification for this cut-off was derived from the overall distribution of ΔFC values for the probes with a |FC| of ≥2 and captures approximately the 10% of extreme ΔFC values for those probes (Figure 2A). Indeed, ΔFC ≥1.3 or ≤1/1.3 probes represent a reasonable selection of probes in terms of differential intensity of triggered probes (Figure 2B). The 50 probes identified represent a total of 44 genes (Table 3). Since all of these genes belonged to the cluster of transcripts that were up-regulated by the *M. leprae* sonicate stimulation, the identified genes differed in their intensity of transcriptional up-regulation. Hence, reference to over-stimulated or under-stimulated genes in T1R patients is always relative to T1R-free leprosy patients.

**Table 3:** List of 44 T1R signature genes differently regulated in former T1R group in comparison with former leprosy patients, sorted by functional group.

Genes symbol	FC_T1R_/FC_LEP_ (ΔFC)^a^	Function
*I. Pro-inflammatory regulators*		
*ADA*	5.57/4.0 (1.39)	Activates T cell proliferation and Th1 cytokines production [40]
*CCL2*	13.96/7.74 (1.80)	Monocyte recruitment [41]
*CCL3*	74.03/54.3 (1.36)	Recruitment of CD8+ T cells [42]
*CCL3L1* *	60.6/44.8 (1.35)	Macrophage and T cell recruitment [43]
*CCL3L3*	26.5/17.4 (1.52)	Monocyte recruitment [44]
*CCL4L1*	11.2/6.7 (1.66)	Monocyte recruitment [41]
*CCL20*	11.15/8.4 (1.32)	Dendritic, activated T cells recruitment [45]
*IL1A*	31.5/21.4 (1.47)	Pro-inflammatory cytokine [46]
*IL1B*	62.0/40.2 (1.54)	Pro-inflammatory cytokine [47], [48]
*IL6*	14.8/11.1 (1.33)	Pro-inflammatory cytokine [47], [48]
*IL23A*	10.85/8.05 (1.35)	Pro-inflammatory cytokine [48]
*TNFAIP2SPP1*	3.4/2.3 (1.48) 2.5/3.5 (0.71)	Pro-inflammatory cytokine [49], [50]; Promotes cell migration [51] Pro-inflammatory cytokine [52]–[54], indicator of host response to mycobacterial infection [55]
*TAGAP* *	3.35/4.67 (0.72)	T-cell activation [56], association with pro-inflammatory diseases [57], [58]
*FCAR*	2.6/3.57 (0.73)	Stimulates cytotoxicity, release of pro-inflammatory cytokines [59]
*II. Arachidonic acid pathway*		
*PTGS2* *	16.8/8.5 (1.98)	Production of PGs [31]
*TNFAIP6*	12.0/6.0 (2.0)	Induction of COX-2 [30]
*GPR109B*	5.9/4.5 (1.32)	Induction of PGs production [60]
*CYP1B1*	4.3/3.1 (1.4)	Metabolism of AA [61]
*C1QTNF1 ADCY3*	2.9/2.1 (1.38) 6.03/8.65 (0.69)	Induction of PGs production [62], [63]Induction of PGs production [62], [63]
*III. Negative regulation of inflammation*		
*ORM1*	95.1/60.6 (1.57)	Strong anti-inflammatory function [32], [64]
*KYNU* *	7.5/5.5 (1.38)	Kynurenine metabolism [37]
*IDO1*	9.9/7.5 (1.32)	Tryptophan metabolism [37]
*PLA2G7*	8.7/6.7 (1.30)	Anti-inflammatory regulator [39]
*PI3*	15.7/8.2 (1.90)	Anti-inflammatory regulator, antimicrobial molecule, tissue repair [65]
*SPINK1 SOD2* *	24.2/15.4 (1.57) 3.2/5.9 (0.54)	Prevents activation of proMMPs [66] Suppressor of oxidative tissue damage [67]; anti-inflammatory effect [68]
*KLF10*	2.0/2.7 (0.74)	Repression of inflammation and proliferation [69]
*LILRA3*	3.4/4.9 (0.69)	Potential anti-inflammatory function, upregulated by IL-10 [70]
*CD274*	4.5/6.0 (0.75)	Regulation of T cells activation and tolerance [71]
*MAFB*	2.4/3.6 (0.67)	Induces monocyte-macrophage differentiation [72], development of autoreactive cell [73]
*IV. Unclassified genes*		
*AGAP3*	3.8/2.7 (1.37)	GTPase
*C20ORF160*	2.76/2.0 (1.37)	Unknown function
*C15ORF48*	29.6/18.4 (1.61)	Unknown function
*MARCKS*	5.6/4.2 (1.33)	Neutrophil adhesion and migration
*NBN*	3.9/2.8 (1.39)	DNA damage repair
*RGL1 FCGR1B*	3.3/2.5 (1.32 ) 3.5/4.6 (0.76)	Regulation of signal transduction via small GTPasesHigh-affinity receptor for immune-globulins
*GNPDA1*	4.2/5.5 (0.76)	Glucosamine catabolic process
*LIMK2*	2.5/3.3 (0.76)	Cell cycle, spindles, chromosomal division
*LRRC50*	2.7/3.8 (0.73)	Assembly of the dynein-arm complexes
*PLEKHB2*	4.8/6.4 (0.75)	Protein binding
*TMEM158*	3.3/5.3 (0.62)	Transmembrane protein

a Expression fold-change in response to *M. leprae* antigens for the T1R patients compared with T1R-free leprosy patients in the retrospective arm (ΔFC = FC_T1R_/FC_LEP_).

* The mean fold-change is provided for genes with multiple probes.

We classified the 44 differentially regulated genes by their functional roles in immunological processes. More specifically, we assigned 32 genes into 3 distinct groups: i) genes promoting a pro-inflammatory response; ii) genes belonging to the arachidonic acid (AA) metabolic pathway, iii) genes involved in down-regulation of the inflammatory response. The remaining 12 genes were not assigned to any specific functional class, i.e. they were regarded as unclassified genes (Table 3). There were larger proportions of under-stimulated genes in the groups of “negative regulation of inflammation” and “unclassified genes” as compared to the groups “pro-inflammatory regulators” and “arachidonic acid pathway” (Table 3). We defined these 44 genes as T1R gene set signature.

### Validation of the T1R gene set in a prospective sample

Approximately two-thirds of T1R patients develop clinical symptoms of T1R only after diagnosis of leprosy and initiation of treatment. Such patients can be enrolled in a prospective design. We collected blood samples from 43 recently diagnosed borderline leprosy patients with no signs of T1R at the time of leprosy diagnosis. Blood stimulation with *M. leprae* sonicate at the time of enrolment was performed by the identical procedure used in the retrospective study. All patients were followed for at least 3 years and a total of 11 individuals with T1R episodes were recorded. After 3 years no additional episodes of T1R are expected to occur [8]. Transcriptome analysis of whole blood assays from the prospective samples detected 752 genes regulated by *M. leprae* sonicate with ≥2-fold change in T1R-affected and/or T1R-free patients. The gene sets and pathways represented by these genes largely overlapped those detected in the retrospective sample (Table S1).

As in the retrospective discovery arm, no single gene was significantly differentially regulated between T1R-free and T1R-affected leprosy patients in the prospective arm. Therefore, we focused our approach on the systematic analysis of differentially expressed groups of genes with special focus on the T1R-specific gene set. To test for the significance of differential regulation of groups of genes between T1R-affected and T1R-free patients we performed receiver operator characteristic scoring (ROC) analysis [23], [24]). The ROC algorithm, an equivalent of the Wilcoxon rank sum test, performs ranking of genes based on their scores. We used the absolute log_2_ transformed ΔFC values as gene scores. Subsequently, ROC clusters genes according to GO terms and user-defined gene sets and tests for the overrepresentation of high scoring genes in each gene-set. As the proportion of top ranking genes in a gene set increases, the set becomes more significant. We performed the initial analysis by comparing T1R patients to T1R-free leprosy patients in the retrospective sample, and validated the results in the prospective sample. Contrary to the approach used for the T1R-specific set in the discovery phase, for the ROC analysis we used all available probes without a specified score cut-off point to avoid bias in the results. We restricted the number of genes representing an individual gene set to be between 5 and 100. As the ΔFC value can have two directions we split the probe sets into “over-regulated” (ΔFC≥1) and “under-regulated” (ΔFC<1) groups of genes. Since the direction of differential stimulation is an important replication criterion, groups of over- or under-stimulated genes from the same gene set were analysed separately.

The T1R gene set included 29 genes that were over-regulated and 15 genes that were under-regulated (Table 3). As expected, in the retrospective discovery sample the set of 29 over-regulated genes was very significantly enriched (*P*_BH_ = 1.07×10^−53^). Importantly, the same set of genes was very significantly replicated in the prospective validation sample (*P*_BH_ = 2.33×10^−9^; Table 4). Next, we analysed the 10 GO terms most significantly overrepresented among T1R patients in the retrospective sample for replication in the prospective sample. As no gene score cut-off was used for ROC analysis the GO terms included up to 100 genes. Several of these GO terms concerned immune cells activation. For example, 72 genes of the GO term “Regulation of leukocyte activation”, were significantly more up-regulated in T1R patients in both the discovery (*P*_BH_ = 2.33×10^−6^) and the validation sample (*P*_BH_ = 4.91×10^−3^; Table 4). Likewise, a set of 97 genes of the lymphocyte activation GO term was significantly stronger regulated in T1R patients in both discovery (*P*_BH_ = 3.28×10^−6^) and replication sample (*P*_BH_ = 3.91×10^−4^). In addition, several replicated GO terms related to control of cellular immune response, including “Regulation of cell activation” (Retrospective *P*_BH_ = 6.88×10^−6^ and Prospective *P*_BH_ = 9.44×10^−3^), “Anti-apoptosis” (Retrospec *P*_BH_ = 1.00×10^−5^ and Prospec *P*_BH_ = 1.74×10^−4^) and “Response to virus” (Retrospec *P*_BH_ = 1.36×10^−5^ and Prospec *P*_BH_ = 5.74×10^−6^; Table 4). Of note, among the ten highly significant GO terms with up-regulated genes in retrospective T1R patients, four did not replicate in the prospective sample (Table 4).

**Table 4:** The T1R signature gene set and GO terms significant differentially regulated by T1R-affected compared to T1R-free leprosy patients-in discovery and validation sets.

GO term	Gene number^a^	RETROSPEC *P*_BH_-value^b^	PROSPEC *P*_BH_-value^c^
*T1R signature gene set*	29	1.07×10^−53^	2.33×10^−9^
Translational elongation	77	4.39×10^−13^	0.01005
Regulation of leukocyte activation	72	2.33×10^−6^	4.91×10^−3^
Nuclear transport	73	2.41×10^−6^	0.01130
Lymphocyte activation	97	3.28×10^−6^	3.91×10^−4^
Positive regulation of immune system	94	5.20×10^−6^	0.02017
Regulation of cell activation	75	6.88×10^−6^	9.44×10^−3^
Protein import	62	1.03×10^−5^	0.02767
Anti-apoptosis	93	1.00×10^−5^	1.74×10^−4^
Response to virus	66	1.36×10^−5^	5.74×10^−6^
Regulation of I-κB/NF-κB cascade	62	5.83×10^−5^	2.60×10^−4^

a Number of genes assigned to a particular gene set in the retrospective sample.

b *P*_BH_-value obtained by ROC analysis of differential gene expression between former affected and T1R-free patients (retrospective sample).

c *P*_BH_-value obtained by ROC analysis of differential gene expression between prospective affected and T1R-free patients (validation sample).

When we analysed the list of 15 genes under-stimulated in the T1R gene set signature in the retrospective arm, we also observed a very significant differential regulation T1R (*P*_BH_ = 7.7×10^−31^) which was expected since the T1R gene set had been derived from the same data. More importantly, this result was replicated in the prospective sample (*P*_BH_ = 2.36×10^−3^; Table 5). Among the ten most significantly under-stimulated GO terms we observed a preponderance of gene sets related to RNA processing the majority of which was replicated in the prospective sample (Table 5). For example, we detected two large gene sets involved in negative regulation of cellular protein metabolic processes (100 genes) and protein modification processes (82 genes) that were significant under-stimulated in T1R patients in both the retrospective and prospective samples (Retrospec *P*_BH_ = 5.98×10^−4^/Prospec *P*_BH_ = 3.97×10^−4^, and Retrospec *P*_BH_ = 1.00×10^−3^/Prospec *P*_BH_ = 1.18×10^−4^, respectively; Table 5). Overall, from 20 tested gene sets we successfully replicated thirteen with the combined T1R gene set showing the strongest evidence for replication for any gene set.

**Table 5:** The T1R signature gene set and GO terms significantly less induced by leprosy patients affected by T1R compared to T1R-free patients-in discovery and validation sets.

GO term	Gene number^a^	RETROSPEC *P*_BH_-value^b^	PROSPEC *P*_BH_-value^c^
*T1R signature gene set*	15	7.70×10^−31^	2.36×10^−3^
RNA localization	45	2.96×10^−5^	4.42×10^−3^
RNA transport	43	3.13×10^−5^	6.76×10^−3^
Posttranscriptional regulation of gene expression	87	7.62×10^−5^	2.55×10^−3^
mRNA transport	39	2.99×10^−4^	0.01169
Protein intracellular transport	39	3.28×10^−4^	7.72×10^−5^
Negative regulation of cellular protein metabolic processes	100	5.98×10^−4^	3.97×10^−4^
Regulation of translation	62	6.14×10^−4^	6.09×10^−5^
Regulation of defense response to virus	14	9.83×10^−4^	0.17832
Negative regulation of protein modification process	83	1.00×10^−3^	1.18×10^−4^
Regulation of I-κB/NF-κB cascade	62	1.62×10^−3^	2.60×10^−4^

a Number of genes assigned to a particular gene set in retrospective sample.

b *P*_BH_-value obtained by ROC analysis of differential gene expression between former affected and T1R-free patients (retrospective sample).

c *P*_BH_-value obtained by ROC analysis of differential gene expression between prospective affected and T1R-free patients (prospective sample).

### Impact of covariates

We evaluated a possible impact of gender and age at diagnosis on the significance of the T1R gene set. In the discovery arm of the study we had equal numbers of males (3) and females (3) among the T1R patients. We tested ΔFC ranking for heterogeneity by gender and found significant over-representation of both over- and under-stimulated genes of the T1R gene set among males (*P*_BH_ = 9.5×10^−20^ and *P*_BH_ = 2.1×10^−10^, respectively). In the prospective arm, only males developed T1R and an impact of gender could not be tested. To test for impact of age, we subdivided subjects into 15 years and younger (n = 3) or older than 15 years (n = 3) and found significantly better ranking of the T1R gene set in older patients for over- but not for under-stimulated genes (*P*_BH_ = 1×10^−14^ and *P*_BH_ = 0.16, respectively). The impact of age on the T1R set was not unexpected since young age (<16 years) is highly protective for T1R in Vietnam [8]. In the prospective sample, only one T1R case was younger than 16 years and no further tests were conducted. While the T1R-gene set ranked significantly in all sub-groups, the data suggested that the T1R gene set might have better characteristics for older male patients. Next, we investigated the impact of time to onset of T1R after leprosy diagnosis on replication of the T1R gene set. First, we compared ΔFC values between four subjects who presented with T1R within one month of leprosy diagnosis and the seven patients that developed T1R more distant to initial diagnosis (2 to 21 months). The ΔFC values for the T1R gene set ranked significantly better among late onset T1R (*P*_BH_ = 1.89×10^−16^ and *P*_BH_ = 1.14×10^−5^ for over-and under-stimulated genes, respectively). These results suggest that rapid onset patients already displayed preclinical forms of T1R and that the onset of T1R immune dysregulation in those patients overwhelmed the predictive signature.

## Discussion

Despite the effective treatment of T1R by corticosteroids, neurological impairment persists in about 30% to 50% of cases [25]. Thus, the early identification of patients at risk of T1R, and consequently at risk of neurological injury, is a critical challenge in leprosy care [26]. The identity of the factors that commit certain leprosy patients to T1R while others remain free of such complications is unknown. Episodes of T1R are characterized by a highly dysregulated inflammatory response which makes it difficult to discern specific functional indicators of T1R risk in patients with ongoing reactions. To avoid this problem, we enrolled former T1R patients that had undergone an episode of T1R at least seven years earlier and compared their reaction to *M. leprae* antigens with the one displayed by former leprosy patients that had remained T1R-free. This sample was too small to assess a possible impact of time since cure on the transcriptional response but the spread of time since cure across all subjects was rather narrow suggesting that this would not bias results. As assay we used transcriptional profiling of whole blood cultures stimulated with high doses of *M. leprae* sonicate. The gene sets that were preferentially regulated in former T1R patients were then validated in a prospective study where we followed newly diagnosed leprosy patients at risk of T1R for three years. Transcriptome responses to *M. leprae* antigens were compared between patients who developed T1R and those who did not. Hence, our study was not designed to study pathogenic networks of host response during T1R but to evaluate characteristics of the transcriptomic response of T1R patients to *M. leprae* antigen prior to the onset of any clinical symptoms of T1R. The important result of our study was that we were able to define a T1R gene set signature based on the magnitude of expression differences in the retrospective sample that we replicated very significantly in an independent prospective sample. This T1R gene set now needs to be investigated for its possible predictive value in follow-up studies.

An important aspect of our analysis was the finding that for individual transcripts we failed to detect significant differences in *M. leprae* antigen triggered gene induction between T1R-affected and T1R-free leprosy patients. The lack of significant differences for individual gene transcripts prompted us to analyse differences of induction in sets of genes involved in the same cellular functions. Gene sets reflect biological pathways or processes and therefore represent a higher level of host responses to stimuli than individual genes. The fact that gene set analyses can identify changes in host responsiveness that cannot be detected through the study of any individual gene is a powerful feature of such analyses. Since our interest was in the difference of gene induction by *M. leprae* antigen between the two groups of patients, we used the difference in gene induction as a “score” to characterize the transcriptional response of individual genes. These gene scores were then used in the gene set analysis. By considering the scores of all genes on the expression chip we were able to identify gene sets that significantly differed between the two patient groups in a global and unbiased fashion. In addition, the genes that are part of a gene set need to have a strong biological link to the phenotype to which the gene set is assigned. A substantial number of genes in the T1R set signature are strong mediators of a pro-inflammatory response which is driven by monocytes and lymphocytes reflecting the clinical picture of T1R episodes which are characterized by excessive cell-mediated immunity [9], [27]. This is best shown by the large number of chemokine-coding genes that are more strongly upregulated in T1R patients since the encoded molecules mediate the recruitment of monocytes and lymphocytes to the site of inflammation (Table 3). For example, identification of *CCL2* as a part of the T1R gene set is not surprising and could be considered as a positive control of the experiment as it is entirely consistent with the reported increase of *CCL2* expression in lesions of active T1R patients [28].

The T1R gene set contains a substantial number of genes involved in arachidonic acid metabolism. The release of AA and its derivatives is a crucial step in the regulation of pro- and anti-inflammatory signalling. For example, *PTGS2*, encoding COX-2, a central gene in the AA pathway, was one of the preferentially upregulated genes in the T1R gene set signature. Clinical detection of increased levels of COX-2 in edemas, vessels and nerves of T1R patients [29] is supported by the drastic upregulation of *PTGS2* in T1R patients. In line with this observation, *TNFAIP6*, which was highly expressed in the early onset T1R samples, encodes TNF-stimulated gene 6 (TSG6), an inducer of COX-2 expression in macrophages [30]. COX-2 oxidizes AA leading to the production of prostaglandins which are powerful mediators of pro- and anti-inflammatory responses [31].

A substantial number of genes in the T1R gene set represent strong mediators of the anti-inflammatory response. For example, alpha-1-acid glycoprotein (AGP), encoded by the *ORM1* gene, is a potent inhibitor of neutrophil chemotaxis and superoxide anion generation [32]. The serum level of AGP is used as a biomarker of leprosy type-2 reactions and Crohn's Disease management [33], [34]. Elafin, coded by *PI3*, can effectively de-activate neutrophil elastase thus preventing excessive tissue damage [35]. Additionally, by inhibiting NF-κB and AP-1 activity elafin controls the extent of the inflammatory response in tissues [36]. *IDO1* and *KYNU* encode the anti-inflammatory regulators indoleamin-2,3-dioxygenase (IDO) and kynureninase (KYNU), respectively, that are involved in the tryptophan metabolic pathway [37]. The metabolites of the tryptophan breakdown represent potent mediators of the anti-inflammatory response [38]. The oxidative action of IDO on tryptophan is dependent on the presence of the superoxide radical anion (O_2_^−^) which is utilized by IDO both as a substrate and a cofactor. In a competitive reaction O_2_^−^ is inactivated by superoxide dismutase (SOD2). Therefore, the observed relative down-regulation of *SOD2* in retrospective T1R patients favours the presence of superoxide radical anions providing both substrate and cofactor for IDO. The coordinated up-regulation of *IDO1* and *KYNU* and down-regulation of *SOD2* reveals a strong anti-inflammatory response in the retrospective T1R patients. Finally, *PLA2G7* encodes a phospholipase A that inactivates the potent pro-inflammatory mediator platelet-activating factor (PAF) [39].

The usefulness and robustness of the T1R gene set was demonstrated by the validation of the T1R set signature in a prospective study. For this, we conducted whole blood assays for newly diagnosed leprosy patients before the onset of clinical symptoms of T1R. The validity of the T1R gene set signature in this design directly demonstrates that T1R patients have an innate predisposition to mount a strong pro-inflammatory response to *M. leprae* antigens despite the up-regulation of major anti-inflammatory genes. Hence, the apparent breakdown of communication between pro- and anti-inflammatory responses in T1R patients appears as characteristic of T1R susceptibility. However, it is important to realize that the T1R gene set does not capture all aspects of predisposition to T1R nor does it allow conclusions about the effector mechanism at work during ongoing T1R. Hence, while our data point to a primary role of innate immunity in predisposition it is possible that acquired immunity responses missed by our design make an important contribution to T1R predisposition. Nevertheless, the results of our experiments raise two immediate questions. What are the genetic factors that predispose a person to undergo T1R after exposure to *M. leprae* antigens and is the observed breakdown in the regulation of pro-inflammatory responses specific to T1R patients? The answers to these questions may impact on a multitude of human inflammatory diseases.

## Materials and Methods

### Ethics statement

The study was conducted according to the principles expressed in the declaration of Helsinki. Informed consent was obtained for all subjects participating in the study. The study was approved by the regulatory authorities and ethics committees in Ho Chi Minh City, Vietnam, and the Research Ethics Board at the Research Institute of the McGill University Health Centre, Montreal, QC, Canada.

### Human subjects

For the retrospective study, we recruited 12 unrelated Kinh Vietnamese subjects at the Dermato-Venereology (DV) Hospital in Ho Chi Minh City, Vietnam. These individuals had previously been diagnosed with borderline leprosy (BT: n = 3, BB: n = 7, BL: n = 2) and six of them had presented with T1R at the time of their leprosy diagnosis. At the time of recruitment for the present study, all individuals had been cured and had remained asymptomatic for at least five years. Males (n = 5) and females (n = 7) were approximately equal distributed across both groups. Age at the time of leprosy diagnosis ranged from 9 to 28 years, with a median age of 18 years. For the prospective study, we recruited 43 individuals recently diagnosed with borderline leprosy without T1R. Blood samples were collected from patients within less than 3 months of their leprosy diagnosis and before undergoing T1R. Enrolled individuals presented with five borderline leprosy subtypes (BT: n = 10, BT/BB: n = 6, BB: n = 19, BB/BL: n = 2, BL: n = 6). Among the recruited patients 34 were males and 9 were females; the median age was 27 years (range 9 to 41 years). The recruitment of younger individuals for the retrospective study is explained by age being a risk factor for the occurrence of T1R at the time of diagnosis [8]. The male∶female ratio is representative of the patient hospital turn in. All prospective patients were closely followed for 3 years during which 11 patients developed T1R.

### Preparation of *Mycobacterium leprae* sonicate

*M. leprae* whole cell sonicate was generated with support from the NIH/NIAID Leprosy Contract N01-AI-25469 at Colorado State University. Inactivated (irradiated) armadillo-derived *M. leprae* whole cells were probe sonicated with a Sanyo sonicator to >95% breakage to produce whole cell sonicate.

### Whole-blood assay

A total of 20 ml of whole blood was obtained from each subject by venipuncture in EDTA vacutainers. Blood samples were split in two aliquots and each aliquot was mixed with RPMI medium containing L-glutamine (300 mg/L) and HEPES (10 mM) at 1∶2. One aliquot was stimulated with *M. leprae* sonicate at a concentration of 20 µg/ml, which approximately corresponds to an MOI of 50 *M. leprae* per white blood cell. The second aliquot was left untreated. Each aliquot, the stimulated one and the control, was divided into four 50 ml polystyrene tubes to facilitate better leukocytes adhesion and aeration of blood. Tubes were incubated for 26–32 hours at 37°C, 5% CO_2_.

### RNA extraction

Total RNA from blood samples was extracted employing a modified protocol of the LeukoLOCK RNA extraction kit (Ambion, CA, USA). Briefly, blood aliquots were filtered by gravity through LeukoLOCK filters to isolate leukocytes. Collected cells were rinsed to eliminate red blood cells and lysed directly on the LeukoLOCK filters. Extraction of total RNA was performed according to the manufacturer's instructions. Isolated RNAs were kept under ethanol and ammonium acetate at −80°C. Prior to further experiments, all samples were cleaned with the RNeasy kit (Qiagen, Germany). The quality of 110 RNA samples (stimulated with *M. leprae* sonicate or not for each of 55 individuals) was assessed by the BioAnalyzer (Agilent). All samples showed RNA Integrity Numbers above 8.5, indicating a good RNA quality, were reverse transcribed, amplified and labelled for hybridization following standard protocol.

### Microarray data analysis

The retrospective 24 samples (12 stimulated and 12 non-stimulated) from healthy controls and former leprosy patients were hybridized to Illumina HumanRef_6_v3 BeadChips and screened for expression changes of 48,804 individual probes (representing 37,804 loci). The prospective 86 samples were hybridized to Illumina HumanHT_12_v4 BeadChips and screened for 47,323 probes (34,695 loci). Raw data were collected by BeadStudio v3.3.7 (Illumina Inc., CA). Utilizing FlexArray 1.6.1.1 (http://genomequebec.mcgill.ca/FlexArray) raw data were subjected to variance-stabilization transformation (VST) and quantile normalization. In the retrospective arm, the regulation of transcription was determined by comparing mean expression values for each probe in stimulated and unstimulated samples of each phenotype group. The expression FC was estimated using the formula: 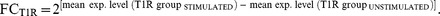 To focus on highly *M. leprae*-regulated transcripts we selected all genes whose expression levels were increased or decreased by a fold-change of 2 or more. To compare the extent of transcription regulation between T1R-affected and T1R-free leprosy patients we looked at the ratio in post-stimulatory expression changes, ΔFC = FC_T1R_/FC_Lep_. We selected genes that were differentially regulated between two groups with ΔFC≤1/1.3 (termed under-regulated) and ΔFC≥1.30 (termed over-regulated). We employed DAVID version 6.7 (http://david.abcc.ncifcrf.gov/; [20], [21]) to estimate the enrichment of Gene Ontology (GO, [22]) terms and metabolic or signalling pathways within the list of genes regulated by *M. leprae* sonicate. We selected the GO terms of levels 3 to 5 which assign more specific functional annotation to each gene. Due to involvement in multiple processes some genes are assigned to multiple GO terms. We used Benjamini-Hochberg correction for multiple testing (*P*_BH_), controlling the false-discovery rate (FDR) at 0.05. A particular GO term was considered significantly overrepresented in a gene list when its *P*_BH_-value was <10^−5^.

### Receiver operator characteristic (ROC) scoring

ROC was performed using ErmineJ software (http://www.chibi.ubc.ca/ermineJ/, [23], [24]). ROC is used as the standard method of evaluating genes scores by their ranking. The algorithm is based exclusively on the order of the underlying values (gene scores). The ROC method tests the null hypothesis that genes represented in gene sets are randomly distributed in their ranking. For each gene set the ROC value is calculated, which reflects the area under the curve, ranging from 0.5 (genes in the set are ranked randomly) to 1.0 (the gene set includes only the highest-scoring genes). Thus, ROC evaluates the probability of high-scoring genes to belong to a specific gene set while accounting for the number of genes in the set. We used absolute values of log_2_ transformed ΔFC values for the probes as gene scores in the ROC algorithm. All available probes were used for the analyses. For genes represented by multiple probes the mean score was used. For the analysis we only considered biological processes GO terms containing 5–100 genes. In addition to the T1R gene set, the ten most significant differentially regulated GO terms in the retrospective arm were tested for replication in the prospective arm.

## Supporting Information

Table S1 Overrepresented Gene Ontology terms and KEGG pathways amongst the 752 genes regulated (|FC|≥2) by *M. leprae* sonicate in whole blood of subjects in the prospective arm.
(DOC)
