# Supplementary material for: Gene Set Signature of Reversal Reaction Type I in Leprosy Patients
Source: PLoS Genet. 2013 Jul 11;9(7):e1003624. doi: 10.1371/journal.pgen.1003624 (PMC3708838; doi:10.1371/journal.pgen.1003624)
Supplement: Table S1 — Overrepresented Gene Ontology terms and KEGG pathways amongst the 752 genes regulated (|FC|≥2) by M. leprae sonicate in whole blood of subjects in the prospective arm. (DOC) [file pgen.1003624.s001.doc]

**Table S1.** Overrepresented Gene Ontology terms and KEGG pathways amongst the 752 genes regulated (│FC│ ≥ 2) by *M. leprae* sonicate in whole blood of subjects in the prospective arm.

| **GO terms** | **Number of genes^a^** | **% input^b^** | ***P*-value^c^** | ***P*_BH_-value^d^** |
| --- | --- | --- | --- | --- |
| Defense response | 87 | 11.9 | 4.52 x 10^-24^ | 3.55 x 10^-21^ |
| Inflammatory response | 59 | 8.1 | 1.67 x 10^-21^ | 2.48 x 10^-18^ |
| Vacuole | 50 | 6.8 | 1.45 x 10^-20^ | 3.87 x 10^-18^ |
| Response to wounding | 75 | 10.3 | 1.01 x 10^-20^ | 3.97 x 10^-18^ |
| Regulation of immune system process | 61 | 8.3 | 2.77 x 10^-19^ | 7.24 x 10^-17^ |
| Regulation of response to stimulus | 63 | 8.6 | 2.78 x 10^-16^ | 6.54 x 10^-14^ |
| Response to other organism | 43 | 5.9 | 1.24 x 10^-12^ | 1.40 x 10^-10^ |
| Regulation of cell proliferation | 77 | 10.5 | 3.32x 10^-12^ | 3.26 x 10^-10^ |
| Chemotaxis | 30 | 4.1 | 1.68 x 10^-11^ | 1.32 x 10^-9^ |
| Response to lipopolysaccharide | 22 | 3.0 | 3.45 x 10^-12^ | 1.70 x 10^-9^ |
| Regulation of response to stress | 39 | 5.3 | 5.72 x 10^-11^ | 3.74 x 10^-9^ |
| Regulation of cytokine production | 31 | 4.2 | 7.68 x 10^-11^ | 4.64 x 10^-9^ |
| Response to bacterium | 32 | 4.4 | 8.43 x 10^-11^ | 4.73 x 10^-9^ |
| Regulation of cell death | 74 | 10.1 | 3.11 x 10^-10^ | 1.44 x 10^-8^ |
| Response to cytokine stimulus | 20 | 2.7 | 3.67 x 10^-10^ | 1.60 x 10^-8^ |
| Cytokine activity | 32 | 4.4 | 1.28 x 10^-10^ | 2.85 x 10^-8^ |
| Regulation of cell activation | 29 | 4.0 | 7.67 x 10^-10^ | 3.01 x 10^-8^ |
| Signal transduction | 174 | 23.8 | 1.83 x 10^-8^ | 4.63 x 10^-7^ |
| Cytoplasmic part | 270 | 36.9 | 3.08 x 10^-9^ | 5.23 x 10^-7^ |
| **KEGG Pathways** | **Number of genes^a^** | **% input^b^** | ***P*-value^c^** | ***P*_BH_-value^d^** |
| Lysosome | 27 | 3.7 | 3.20 x 10^-9^ | 2.47 x 10^-7^ |
| Cytokine-cytokine receptor interaction | 42 | 5.7 | 7.15 x 10^-9^ | 3.67 x 10^-7^ |
| Chemokine signaling pathway | 33 | 4.5 | 4.34 x 10^-8^ | 1.67 x 10^-6^ |
| Epithelial cell signaling in  *H. pyroli* infection | 18 | 2.5 | 3.11 x 10^-7^ | 9.57 x 10^-6^ |
| ^a^ Number of genes assigned to one GO term or pathway.  ^b^ Percentage of genes assigned to one GO term or pathway from the total number of processed genes.  ^c^ *P*-value derived from a modified Fisher’s exact test.  ^d^ *P*-value corrected for multiple testing by Benjamini-Hochberg correction. | | | | |
